# A dataset of modern and fossil distribution of coccolithophore species *Florisphaera profunda* in the world׳s ocean

**DOI:** 10.1016/j.dib.2018.12.079

**Published:** 2019-01-07

**Authors:** I. Hernández-Almeida, B. Ausín, M. Saavedra-Pellitero, K.-H. Baumann, H.M. Stoll

**Affiliations:** a ETH, Geological Institute, Department of Earth Science, Sonneggstrasse 5, 8092 Zürich, Switzerland; b University of Bremen, Department of Geosciences, Klagenfurter Str. 2-4, 28359 Bremen, Germany

## Abstract

We compiled modern and fossil relative abundance of coccolithophore species *Florisphaera profunda* from published and unpublished datasets, along with ocean environmental variable data from satellite remote sensing and physical measurements. The database includes relative abundances of *F. profunda in* sediment trap (*n *= 26) and core-top (*n* = 1258), and sediment core samples (*n* = 104). Downcore data covers the Last Glacial Maximum (*n* = 94, 24-19 ka) or the Mid-to-Late Holocene (*n* = 77, <6 ka). This database allows studying modern and past biogeography of *F. profunda* as a response to changing ocean and climate conditions, “Quantitative reconstruction of primary productivity in low latitudes during the last glacial maximum and the mid-to-late Holocene from a global Florisphaera. profunda calibration dataset” (Hernández-Almeida et al., 2018).

**Specifications table**

**Table:** 

Subject area	*Earth sciences*
More specific subject area	*Marine micropaleontology*
Type of data	*Tables*
How data were acquired	*Public data repositories, Microscope, Digitization*
Data format	*Raw*
Experimental factors	*Samples from unpublished datasets were prepared using standard filtering technique for scanning electron microscope*
Experimental features	*At least 500 coccolithophores were counted at species level at ×3000 magnification in samples from unpublished datasets.*
Data source location	North and South Atlantic Ocean, Indian Ocean.
Data accessibility	*Data are with this article*
Related research article	*Hernández-Almeida I., Ausín, B., Saavedra-Pellitero, M., Baumann, K.-H., Stoll, H.M., 2018 Quantitative reconstruction of primary productivity in low latitudes during the last glacial maximum and the mid-to-late Holocene from a global Florisphaera. profunda calibration dataset. Quat. Sci. Rev, accepted,*[1].

**Value of the data**

• The data of the abundance of *Florisphaera profunda* in modern (sediment trap and surface sediment) allows assessing the environmental factors controlling its biogeography globally.
• The calibration model between relative abundance of *F. profunda* in modern samples and the environmental data, when applied to downcore data, allows reconstructing quantitatively past ocean conditions, such as net primary productivity.
• The data on the downcore abundance of *F. profunda* reveal paleoceanographic changes in the oceans between the Last Glacial Maximum and the Mid-to-Late Holocene.

## 1 Data

*Florisphaera profunda* is a deep-dwelling coccolithophore species which is dominant in tropical and subtropical oceans, whose relative abundance is modern and fossil samples can be used to reconstruct quantitatively environmental conditions in the past [1]. We present data files with information on the relative abundance (%) of coccolithophore species *F. profunda* in selected sediment trap (*n* = 26, Table S1) and surface sediment (*n* = 1258, Table S2) samples around the world׳s oceans, and also in sediment cores (*n* = 104, Tables S3 and S4) covering at least one or both of the following climatic intervals, the Last Glacial Maximum (24–19 ka) and the Mid-to-Late Holocene (<6 ka). Moreover, ocean environmental variables data from gridded satellite remote sensing and physical measurements, interpolated to the location of the modern samples, are also presented.

## 2 Experimental design, materials, and methods

Data of *F. profunda* relative abundance and related metadata (e.g. depth, age, etc.) are mainly from published literature, obtained from public data repositories (PANGAEA), data tables in the publication, sent by the authors. Digitization was performed using the free license software *xyscan* (version 4.3.0). Information about sample preparation techniques for the published datasets can be found in the original publications. *F. profunda* abundance data presented as flux, counts or per gram was transformed to relative abundance (percentage). When several morphotypes of *F. profunda* were identified in the original publication (e.g. *F. profunda* var. *elongata*; *F. profunda* var. *profunda*), these were merged into a single *F. profunda* category. Some unpublished sediment trap (*n* = 93; Atlantic Ocean, 0°–33° N), and downcore (*n* = 9; Atlantic and Indian Oceans, 0°–30° N), datasets are also shown here. These samples were prepared according to standard filtering techniques for coccolithophore counting using scanning electron microscope (SEM), for sediment trap [2] and fossil [3] material. Sediment trap samples were prepared sieving a split of the original sample (between 1/250 and 1/6400) onto a polycarbonate membrane with 0.45 μm pore size. Fossil samples were sieved using filters with the same pore size. Both sediment trap and fossil samples were analysed using a Zeiss DSM 940A SEM at ×3000 magnification, with at least 500 coccolithophores identified at species level.

Annual ocean environmental data for nutrients, sea surface temperature, photosynthetic active radiation, mixed layer depth were interpolated to the location of the surface sediment and sediment trap samples using global 1°×1° resolution datasets from the World Ocean Atlas 2013 [4], [5], [6], [7]. Nutrient and SST data were extracted at 10 and 150 m depth. For the estimation of the ocean primary productivity (NPP), we used MODIS based estimates using the standard vertically Generalized Production Model (VGPM) with a 0.16°×0.16° resolution (https://www.science.oregonstate.edu/ocean.productivity), and also interpolated to the location of the modern samples (surface sediments and sediment traps). For the surface sediment samples, we calculated the Npp based on the mean of the satellite observations available, between 2003 and 2016. The Npp for the sediment trap samples was calculated averaging the same months and year of sampling if the sediment trap was deployed after 2003. For sediment trap samples deployed before 2003, we calculated the monthly mean of the available years and used these data to match up with the same sampling interval of the sediment trap.

## Appendix A Supplementary materials

The following KML files contain the Google maps of the most important areas described in this article.

Map KML file containing the Google map.

## Appendix B Supplementary material

Table S1. Sediment trap *F. profunda* % and monthly Npp dataset. Table S2. Surface sediment *F. profunda* % and modern environmental variables dataset.Table S3. Metadata information of the sediment cores. Table S4. Downcore *F. profunda* % dataset.
